# Pathogenicity and Immune Responses of *Aspergillus fumigatus* Infection in Chickens

**DOI:** 10.3389/fvets.2020.00143

**Published:** 2020-03-11

**Authors:** Zhimin Cheng, Mengxuan Li, Yao Wang, Tongjie Chai, Yumei Cai, Ning Li

**Affiliations:** ^1^College of Animal Science and Technology, Shandong Agricultural University, Taian, China; ^2^Sino-German Cooperative Research Centre for Zoonosis of Animal Origin Shandong Province, College of Animal Science and Technology, Shandong Agricultural University, Taian, China; ^3^Shandong Provincial Key Laboratory of Animal Biotechnology and Disease Control and Prevention, Shandong Agricultural University, Taian, China; ^4^Shandong Provincial Engineering Technology Research Center of Animal Disease Control and Prevention, Shandong Agricultural University, Taian, China

**Keywords:** *Aspergillus fumigatus*, chicken, pathogenicity, TLR signaling, pro-inflammatory cytokines

## Abstract

*Aspergillus fumigatus* is a ubiquitous pathogen in poultry farms, causing aspergillosis in chickens. To study the pathogenicity of *A. fumigatus*, 14-days-old chickens were infected with fungal conidia (2 × 10^7^ CFU/mL) via thoracic intra-air sacs inoculation. The clinical symptoms, gross and histopathological lesions, and fungal load in the lungs were examined. Additionally, the mRNAs of Toll like receptors (TLR) and pro-inflammatory cytokines were evaluated by quantitative PCR to explore the immune responses induced by *A. fumigatus*. The results showed that overt depression, ruffled feathers, and dyspnea were observed in the infected chickens as early as 3 days post infection (dpi). Eleven out of 25 infected chickens died from 5 to 9 dpi, and *A. fumigatus* could also be reisolated from the infected lung. Histopathological examination revealed obvious airsacculitis and pneumonia, characterized by inflammatory cell infiltration (heterophils and macrophages), and granulomatous lesions in the lung. The mRNA expressions of TLR1 and TLR2 were upregulated in the lung and spleen, and most pro-inflammatory cytokines including IL-1β, Cxcl-8, TNF-α, IL-12, and IFN-γ were increased in both the lung and spleen during the tested period, suggesting that the innate immune responses were triggered by *A. fumigatus* infection, and these cytokines participated in the inflammatory responses against *A. fumigatus*. These results indicate that *A. fumigatus* infection by thoracic intra-air sacs inoculation can cause severe respiratory damage in chickens, activate TLR1 and TLR2 mediated immune responses, and elicit large expression of pro-inflammatory cytokines such as IL-1β, Cxcl-8, and IFN-γ. These data will help further understanding of the pathogenesis and immune responses of *A. fumigatus* infection in the chicken.

## Introduction

Avian Aspergillosis is an infectious fungal disease characterized mainly by respiratory symptoms. This disease has been reported worldwide in a large number of wild and domestic birds. Almost all birds are susceptible to aspergillosis, such as chickens (1, 2), turkeys (3), ducks (4, 56), pigeons (5), quails (6), and many wild birds (7, 8). *Aspergillus fumigatus* is one of the most common etiologic agent of aspergillosis. This filamentous fungus is a ubiquitous, opportunistic pathogen that produces large amounts of small-sized conidia in the air. The clinical signs of susceptible poultry differ from flock to flock, age to age and also to the exposure level. Young birds appear to be more susceptible to acute aspergillosis which is characterized by dyspnoea, gasping, and inappetence, resulting in high morbidity and mortality, thus inducing significant economic losses in poultry (9). The chronic form of aspergillosis is sporadic, which generally occurs in older birds, especially breeders in poultry, and causes lesser mortality (10).

Nowadays, aspergillosis is still prevalent in chickens. Sultana et al. detected a total of 912 sick and dead commercial broilers collected from 20 farms at Chittagong district of Bangladesh in 2013. The overall incidence of aspergillosis was found to be 6.14% (11). Since 2007, outbreaks of chicken airsacculitis have occurred in most areas of China, leading to enormous economic losses, feed-borne *A. fumigatus* is the main cause for this respiratory disease (12). Additionally, co-infection of *A. fumigatus* and other pathogens has also been reported clinically (13). Experimental aspergillosis has been studied in chickens for many years (14–16). Recently, Thierry et al. also reported that the conventional JA657 broilers were more susceptible to *A. fumigatus* than the White Leghorn PA12 layers, indicating the lineage of chicken plays an important role in the pathogenicity of *A. fumigatus* (17). The immunopathogenesis of *A. fumigatus* infection in chickens has yet to be fully elucidated.

Host innate immunity is essential for the control of *A. fumigatus*. Many studies regarding the immune response to *A. fumigatus* in human beings and mice have been conducted (18–20), but the immune responses of chickens infected with *A. fumigatus* have not been fully explored. Multiple pattern recognition receptors (PRRs) are involved in recognizing *A. fumigatus*, especially Toll-like receptors (TLR), and C-type lectin receptors (CLR). Furthermore, different components of fungi cell walls can be sensed by different PRRs. It has been confirmed that TLR2 and TLR4 are involved in sensing fungal DNA and zymosan (21, 22). TLR4-deficient mice have a higher susceptibility to *A. fumigatus* compared with control mice (23). TLR2 signaling is essential for responses to *A. fumigatus* in both mouse and human cells (24). C-type lectin receptor, dectin-1, recognizes β-Glucan of *A. fumigatus* in mice alveolar macrophages, and is required for the induction of alveolar macrophage pro-inflammatory responses to *A. fumigatus* (25). Upon recognition, the downstream immune responses mediated by PRRs are triggered, and pro-inflammatory cytokines, such as TNF-α, IL-1β, IL-6, and chemokine Cxcl-8, can be induced to participate in the defense against *A. fumigatus* (26, 27). Conversely, *A. fumigatus* escapes the host immune via modulation or suppression of the relevant signaling pathways (28, 29). As is well-known, the physiologic and anatomic characteristics of chicken respiratory tract are significantly different from that of mammal, and innate immune system is different, such as chicken TLR21 can sense CpG DNA instead of mammalian TLR9 (30). Thus, the pathogenicity and the immune responses of *A. fumigatus* in chickens may also be different.

In the present study, the aim is to investigate the pathogenicity and innate immune responses of *A. fumigatus* in chickens challenged by intra-air sac inoculation. The mortality, clinical signs, gross lesions, and pathological lesions of the infected chickens were observed. Moreover, the expression profiles of innate immune-related genes at 1, 3, and 5 days post infection (dpi) were measured to evaluate the defense against *A. fumigatus*. These results will provide a better understanding of the pathogenesis of *A. fumigatus* and type of immune responses induced in the chicken.

## Materials and Methods

### Strain

*Aspergillus fumigatus* strain (CCCCMIDA1) was purchased from the Institute of Dermatology and Venereology of the Chinese Academy of Medical Sciences and grown on Potato Dextrose Agar (PDA) medium (Solarbio, Beijing, China) for 5–7 days at 37°C according to other report (31). The cultures were washed using sterile phosphate buffer saline (PBS) to collect conidia, and the suspension was filtered through sterile gauze to remove hyphae. The filtered suspension was transferred to an autoclaved centrifuge tube and centrifuged for 15 min at 1,500 × g at room temperature. *A. fumigatus* was re-suspended with sterile PBS to a concentration of 2 × 10^7^ colony forming unit (CFU)/mL.

### Experimental Design

Ten-days-old specific pathogen free (SPF) White Leghorn chickens were purchased from the Poultry Institute, Shandong Academy of Agricultural Science, and housed in isolators with a 12 h photoperiod and provided sufficient water and feed without antibiotics throughout the experiment. Temperature was maintained between 21 and 25°C and relative humidity was 30–40%. When chickens were 14 days old, they were randomly divided into two groups, with 25 in each group. In the infected group, 0.1 mL (2 × 10^6^ CFU) of conidia suspension was inoculated into the right thoracic air sacs of each chicken as previous study (15). Chickens in the control group were inoculated in the same manner with 0.1 mL of sterile PBS. Clinical signs, gross and microscopic lesions, and mortality were observed. Aside from the dead chickens, three live chickens were randomly selected from each group, and the right tissues samples (right lung and air sacs) were collected for histopathological analysis and detection of innate immune-related genes mRNA at 1, 3, and 5 dpi. The rest of the chickens were observed for clinical signs for 9 days and then euthanized at the end of the experiment.

### Lesion Scores of Lungs and Air-Sacs

At necropsy, gross lesions in lungs and air sacs were observed. Lung and spleen were collected and fixed with 4% paraformaldehyde solution to make paraffin sections, which were stained with hematoxylin and eosin for histological examination. The severity of gross lesions and histopathological lesions of infected air sacs and lungs were observed and scored, using the criteria reported elsewhere (12). Briefly, based on the thickness, turbidity, and inflammatory exudate, the severity of gross lesions of air sacs was scored on a scale of 0 to 4: 0, normal, clean, transparent; 1, slightly thickened and turbid or individual local yellow white exudate; 2, yellow white exudate in a few areas of the air sacs; 3, the majority of the air sacs are covered with yellow-white caseous exudate, thick; 4, thick yellow-white exudates are obvious on the thoracic cavity and abdominal cavity. Similarly, gross lesions of the lung were scored on a scale of 0 to 4: 0, normal, faint red; 1, slight edema and hyperaemia; 2, moderate edema, focal necrosis in a few areas of the lung; 3, yellow white necrosis in half areas of the lung; and 4, necrosis in most aeras of the lung, serious congestion.

The scoring criteria for microscopic lesions of the infected lung were as follows. 0, none; 1, edema and hyperaemia of the alveolar wall; 2, inflammatory cell infiltration in the limited areas of the lung; 3, granuloma, large amount of inflammatory cell infiltration, slight necrosis in the lung; and 4, granuloma and severe cell necrosis in the lung.

### *Aspergillus fumigatus* Load in the Lung of the Infected Chickens

A plate count method was adopted to measure the load of *A. fumigatus* in the lung of the infected chicken. In brief, the infected lungs were collected under sterile condition. The 0.1 g sample was mixed with 900 μL of sterile PBS, and the mixture was ground into homogenate. The homogenate of the lung tissue underwent a 10 times dilution until the concentration reached 10^−4^. The 100 μL sample of each dilution was selected to add into the PDA medium, and then cultured for 24 h at 37°C. Plates with between 30 and 300 colonies were considered to be effective. The CFU was counted according to the following formula, and each dilution was performed in triplicate.

CFU/g = the number of colonies in each plate × the dilution ratio × 10^2^.

### Quantitative Real-Time PCR

Total RNA was extracted from the lung and spleen (0.1 g) using the TRIzol Reagent (Takara, Dalian, China) according to the manufacturer's instructions. The RNA concentration was measured and 1 μg RNA was reverse transcribed with HiScript II QRT SuperMix for qPCR (+gDNA wiper) (Vazyme, Nanjing, China). The synthesized cDNA was stored at −20°C until analysis. Primers (Table 1) required in the study were designed using the Primer 3 online software (http://bioinfo.ut.ee/primer3-0.4.0/) based on the published GenBank sequence or refer to other study (32). Quantitative real-time PCR (qPCR) was prepared in 20 μL according to the operation instruction (Vazyme, Nanjing, China) and performed using Roche LightCycler 96 (Roche, Basel, Switzerland). qPCR was performed at 95°C for 30 s, followed by 40 cycles of 95°C for 10 s, and 60°C for 34 s. The dissociation curves were identified at the final step. All samples were amplified in triplicate.

**Table 1:** Primers used in the study.

**Primer**	**Sequence (5^**′**^-3^**′**^)**	**Product size (bp)**	**GenBank No**.
TLR1 F	GCTGTGTCAGCATGAGAGGA	238	AB109401.1
TLR1 R	GTGGTACCTCGCAGGGATAA		
TLR2 F	GAA AGTTCCCCCTTTTCCAG	246	AB046119.2
TLR2 R	AGAGTGCAGAAGGTCCCTGA		
TLR4 F	GTCTCTCCTTCCTTACCTGCTGTT	187	KP410249.1
TLR4 R	AGGAGGAGAAAGACAGGGTAGG		
TLR21 F	GCAGCTCAGCCGCTCTTTT	80	NM_001030558.1
TLR21 R	CCTTCTTCTTCCTCCTCCTCTCC		
IL-1β F	TACACCCGCTCACAGTCCTT	323	DQ393267.1
IL-1β R	AGGCGGTAGAAGATGAAGC		
IL-2 F	CCGTGGCTAACTAATCTGCTG	125	AF000631.1
IL-2 R	AACGTACATTTTGAGCCCGTA		
IL-6 F	TCTGTTCGCCTTTCAGACCTA	142	AJ309540.1
IL-6 R	GACCACCTCATCGGGATTTAT		
IL-12 F	TGAAGGAGTTCCCAGATGC	152	AY262752
IL-12 R	CGTCTTGCTTGGCTCTTTATAG		
IL-18 F	AGCGTCCAGGTAGAAGATAA	209	NM_204608.1
IL-18 R	TCCTCAAAGGCCAAGAAC		
Cxcl-8 F	GCTCTGTCGCAAGGTAGGAC	115	DQ393272.2
Cxcl-8 R	GCGTCAGCTTCACATCTTGA		
TNF-α F	CGCTCAGAACGACGTCAA	116	MF000729
TNF-α R	GTCGTCCACACCAACGAG		
IFN-γ F	GACGGTGGACCTATTATTGT	195	NM_205149.1
IFN-γ R	CACCTTCTTCACGCCATCAG		
β-actin F	CCTCTCTGGCAAAGTCCAAG	200	L08165
β-actin R	CATCTGCCCATTTGATGTTG		
GAPDH F	AGAACATCATCCCAGCGTCC	133	NM_204305.1
GAPDH R	CGGCAGGTCAGGTCAACA		

*The relative fold change of the target gene normalized to each internal reference gene was calculated respectively, and then the geometric mean of the two values was used as the normalized result of the double internal reference genes*.

### Statistical Analysis

The relative expression of the target genes in the infected and control groups was calculated with the 2^−ΔΔCt^ method and expressed as the mean fold changes. The data were presented in terms of relative mRNA expressed as means ± standard deviations (SD). One-way ANOVA method with Duncan's multiple range test was used for evaluating data using GraphPad Prism 5 software (GraphPad Software Inc. USA). *P* < 0.05 were considered to be significant, and values <0.01 were highly significant.

## Results

### Clinical Signs and Gross Lesions of the Infected Chickens

**Figure 1:**
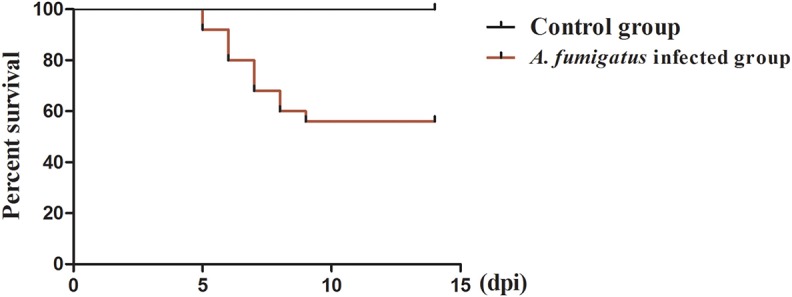
Survival curve after infection with *Aspergillus fumigatus*. Infected chicken was infected with 2 × 10^6^ CFU condia via intra-right thoracic air sac. Control chicken was inoculated with 0.1 ml PBS via the same route. In the *Aspergillus fumigatus* infected group, a total of 11 chickens died from 5 to 9 dpi, respectively. No chickens died in the control group throughout the experiment.

In the *A. fumigatus*-infected group, two chickens died at 5 dpi, three died at 6 and 7 dpi, respectively, two at 8 dpi and one died at 9 dpi. A total of 11 chickens infected with *A. fumigatus* died at 5–9 dpi (Figure 1). Most infected chickens showed obvious clinical signs, including dyspnea, depression, ruffled feathers, and dyskinesia as early as 3 dpi. No chickens died in the control group throughout the study.

The primary organs with gross lesions were the air sacs and lungs, especially the right tissues, the lesions gradually increased with the extension of infection (Table 2). Slightly turbid and local yellow white exudate were observed in two chicken at 1 dpi (Supplementary Figure 1A), and two chickens showed yellow white caseous exudate in a few areas of the air sacs at 3 dpi (Supplementary Figure 1B). However, these lesions increased at 5 dpi. The majority of air sacs were covered with yellow white caseous necrosis exudate in all three infected chickens (Supplementary Figure 1C) compared to that of the control group (Supplementary Figure 1D). For lungs, slight edema, and hyperaemia were observed at 1 dpi (Supplementary Figure 1E), and moderate edema and focal necrosis of the lung were observed at 3 dpi (Supplementary Figure 1F). Diffuse edema and miliary yellow white necrosis were observed in lungs of two infected chickens at 5 dpi (Supplementary Figure 1G). The control lungs were faint red without any lesions (Supplementary Figure 1H). In addition, mild lesions could be observed in other organs including the liver, small intestine, spleen, and skin in the infected group at 3 or 5 dpi.

**Table 2:** Gross lesions scoring of the right infected air sacs and lungs.

**Organs**	**Air sacs**					**Lungs**				
**dpi/score**	**0**	**1**	**2**	**3**	**4**	**0**	**1**	**2**	**3**	**4**
1	1/3^a^	2/3				1/3	2/3			
3	1/3		2/3					3/3		
5				3/3					1/3	2/3

a *Number with lesions of the three chickens examined*.

### Histopathological Lesions of Lungs

**Figure 2:**
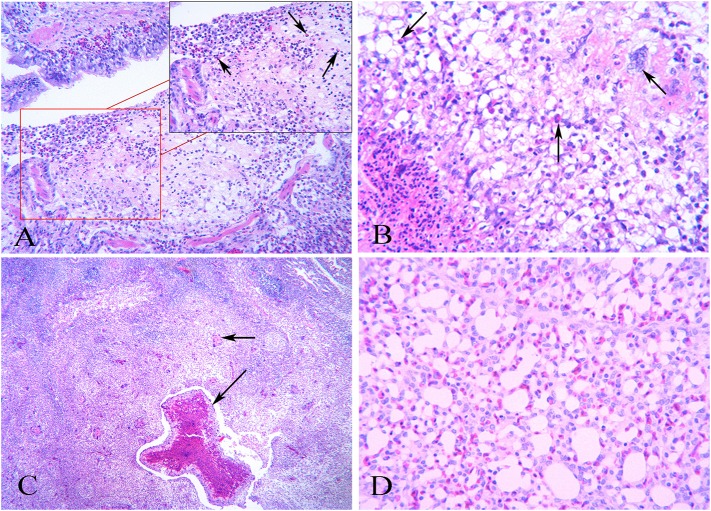
Histopathology of the lungs after infection with *Aspergillus fumigatus*. Right lungs were sampled in infected and control chickens. **(A)** At 1 dpi, inflammatory cell infiltration in the parabronchi. Heterophils can be seen (as indicated by the arrow); **(B)** At 3 dpi, obvious granulomatous inflammation. Arrows indicate inflammatory cells such as heterophils and multinucleated giant cells surrounding the central necrotic area; **(C)** At 5 dpi, progressive inflammation lesions characterized by small granuloma coalescence in the parabronchi (as indicated by the arrow) and massive inflammatory cell infiltration; **(D)** The normal histology structure of chicken lungs in the control group.

The lesions scoring of the right lungs of *A. fumigatus* infected chickens is shown in Table 3. The development of the lesions was gradually becoming more severe from 1 to 5 dpi. The lesions were characterized by inflammatory cell infiltration and granuloma. As shown in Figure 2A, a large number of inflammatory exudates were observed in the parabronchi as early as 1 dpi, including some heterophilic granulocytes; at 3 dpi, the typical granulomatous lesions emerged, characterized by a center of necrotic cells and some fungal elements surrounded by a cliff of heterophils, epithelioid cells, macrophages, multinucleate giant cells, and lymphocytes. Phagocytised fungal elements are observed in the eosinophilic cytoplasm of multinucleated cells (Figure 2B). At 5 dpi, more extensive lesions caused by small granuloma coalescence resulted in parabronchial obliteration with necrotic material, such as degenerated heterophils, and exfoliated epithelial cells (Figure 2C). Normal lung morphology was observed in the control group (Figure 2D).

**Table 3:** Histopathological lesions scoring of the right infected lungs.

**dpi/score**	**0**	**1**	**2**	**3**	**4**
1		2/3^a^	1/3		
3			2/3	1/3	
5				2/3	1/3

a *Number with lesions of the three chickens examined*.

### *Aspergillus fumigatus* Load in the Lung

**Figure 3:**
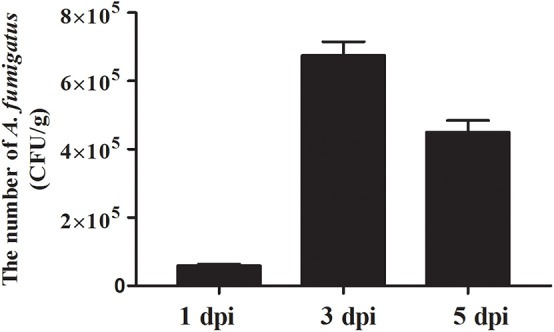
Pathogen load after infection with *Aspergillus fumigatus*. Right lungs were sampled in infected and control chickens (3 chickens per group). *Aspergillus fumigatus* load was measured using the plate counting method and expressed as means CFU per gr of tissue ± SD (*n* = 3).

To determine whether the lung lesions were resulted from *A. fumigatus* infection, the right lungs were collected to measure the fungus load, as the chickens were inoculated via the right thoracic air sacs. As shown in Figure 3, the load of fungus was 5.90 × 10^4^ CFU/g as early as 1 dpi, and reached the peak at 3 dpi, with a value as high as 6.75 × 10^5^ CFU/g. Then the load showed a decreasing trend, and reduced to 4.50 × 10^5^ CFU/g at 5 dpi. These results indicated that *A. fumigatus* was the cause of these lesions.

### Expression of TLR Genes in the Lung and Spleen

**Figure 4:**
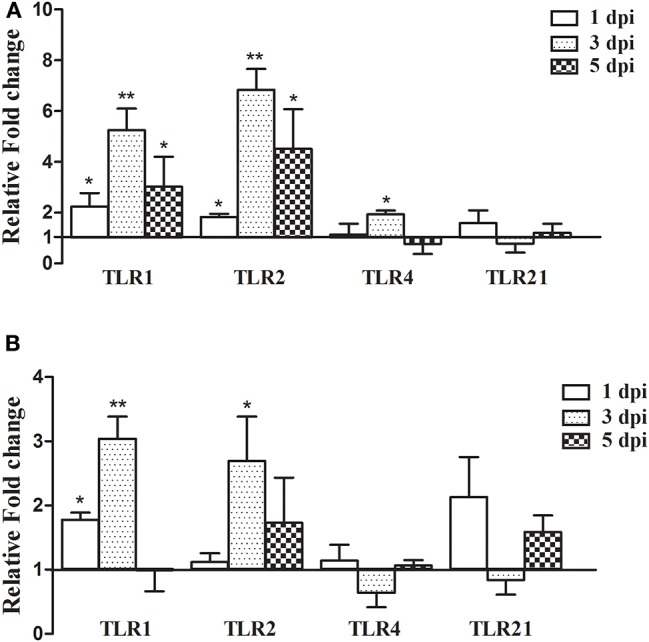
TLR gene expression in the lungs and spleen after infection with *Aspergillus fumigatus*. Spleen and right lungs were sampled at 1, 3, and 5 dpi. Gene expressions in infected chickens were expressed as fold change compared to the control group [means ± SD (*n* = 3)]. β-actin and GAPDH were used as reference genes. **(A,B)** were the expression of TLR gene in the lungs and spleen, respectively. The Y-axis indicates that the relative fold changes of the target gene expression in the *Aspergillus fumigatus* group vs. those in the control group. *p < 0.05, **p < 0.01.

In order to determine the expression of TLR genes after *A. fumigatus* infection, the expressions of TLR1, TLR2, TLR4, and TLR21 were detected in the lung and spleen at 1, 3, and 5 dpi. As shown in Figure 4A, in the lung, the expressions of the TLR1 and TLR2 were upregulated at 1 dpi, the expression peaked at 3 dpi, with the fold change of the TLR2 mRNA being the highest (6.83-fold, *P* < 0.01; Figure 4A), followed by TLR1 (5.25-fold) and TLR4 (1.93-fold). In the spleen, only the expression of TLR1 upregulated compared to the control group at 1 dpi. TLR1 and TLR2 transcripts were significantly increased by 3.04- and 2.70-fold at 3 dpi, respectively (*P* < 0.05; Figure 4B). The expressions of TLR4 and TLR21 has no significantly change (Figure 4B). Collectively, these data indicated that TLR genes were modulated by *A. fumigatus*, especially TLR1 and TLR2, which might be involved in the recognition of *A. fumigatus*.

### Expression of Cytokine Genes in the Lung and Spleen

**Figure 5:**
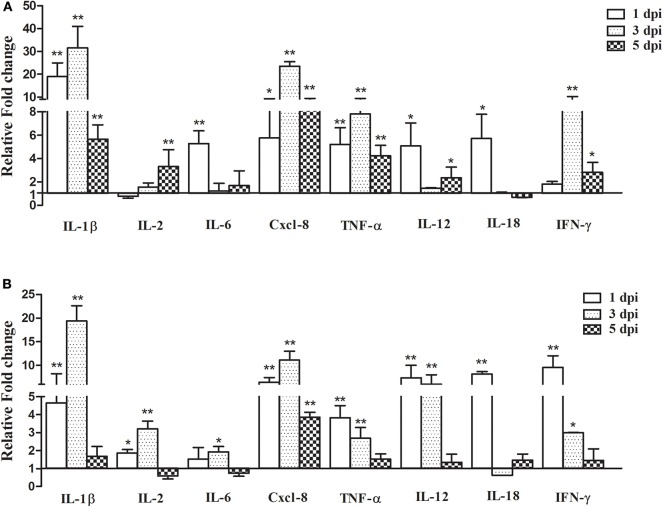
Pro-inflammatory cytokine gene expression in the lungs and spleen after infection with *Aspergillus fumigatus*. Spleen and right lungs were sampled at 1, 3, and 5 dpi. Gene expressions in infected chickens were expressed as fold change compared to the control group [means ± SD (*n* = 3)]. β-actin and GAPDH were used as reference genes. **(A,B)** were the expression of cytokine gene in the lungs and spleen, respectively. The Y-axis indicates that the relative fold changes of the target gene expression in the *Aspergillus fumigatus* group vs. those in the control group. *p < 0.05, **p < 0.01.

To better understand the expression of the downstream pro-inflammatory cytokines in chickens infected with *A. fumigatus*, IL-1β, IL-2, IL-6, Cxcl-8, TNF-α, IL-12, IL-18, and IFN-γ were examined. As shown in Figure 5A, the expressions of most cytokines were upregulated in the lung. At 1dpi, IL-1β, IL-6, Cxcl-8, TNF-α, IL-12, and IL-18 increased significantly. In particular at 3 dpi, the fold changes of the IL-1β, Cxcl-8, TNF-α, and IFN-γ mRNA expression were highly significant with highest value (*P* < 0.01), and the expressions were decreased at 5 dpi. The expression of IL-2 was downregulated at 1 dpi, and then upregulated by 3.32-fold (*P* < 0.01) at 5 dpi. IL-12 and IL-18 expressions increased significantly by 5.08- and 5.71-fold, respectively at 1 dpi, and then decreased in the following 2 days.

In the spleen, the expressions of IL-1β, IL-2, Cxcl-8, TNF-α, IL-12, and IFN-γ were increased at 1 and 3 dpi, but the upregulation decreased at 7 dpi except for IL-2. Most cytokines transcripts reached the highest value at 1 or 3 dpi (Figure 5B). For example, at 3 dpi, IL-1β and Cxcl-8 increased by 19.39- and 11.12-fold, respectively (*P* < 0.01), and the expression levels of TNF-α, IL-12, IL-18, and IFN-γ were highest at 1 dpi, increased by 3.83-, 7.36-, 8.16-, and 9.57-fold, respectively.

## Discussion

*Aspergillus fumigatus*, mainly present in mildewed feedstuff and contaminating ambiant air, is an opportunistic pathogen for poultry inducing respiratory diseases and immunosuppression. The conidia (2.0–3.0 μm in diameter) are easily inhaled and deposited in the lower respiratory tract (33). In the current study, right thoracic intra-air sacs injection was adopted as the inoculation route to infect the 14-days-old SPF White Leghorn chickens with *A. fumigatus* conidia (2 × 10^6^ CFU per chicken). Eleven out of 25 infected chickens died, and most chickens infected with *A. fumigatus* showed overt clinical signs, such as depression, dyspnea, and ruffled feathers. In contrast, the aerosol-challenged layer chickens belonging to White Leghorn lineage showed resistance to this fungus (17). The mechanisms of induction and the type of immunity are different when pathogens by-pass the upper respiratory tract, which would determine the level of resistance or susceptibility to a pathogen. Considering the different routes of inoculation, aerosol infection can quickly activate the mucosal immunity of chickens' upper respiratory tract and thus inducing resistance fungal infection. However, the intra-air sacs inoculation route bypass the upper airways and their associated defense mechanisms. Consequently, *A. fumigatus* infection can be established by this route in the chicken. Indeed a large number of *A. fumigatus* was reisolated from the infected lungs indicated the successful infection. The lesions of the infected chickens induced by this route were mostly compatible with those found in the field cases, that are characterized by pneumonia and airsacculitis (34, 35).

The respiratory tract is an essential infection route of *A. fumigatus*. In our experiment, the necropsy analysis demonstrated that the severe gross lesions were mainly on the air sacs and lungs, though the small intestine, liver, and spleen were also involved. The apparent yellowish-white caseous exudate and necrosis foci were observed in the air sacs and lungs of the infected chickens, especially the right lungs and air sacs which showed more severe lesions. Further histopathological analysis revealed substantial inflammatory cell infiltration in the lung at 1 dpi. Subsequently, typical granulomas lesions were observed in the lung, with a central core of necrotic cells and fungal elements, and a rim of the epithelioid cells, macrophages, heterophils, and multinucleate giant cells. These gross and microscopic lesions of the infected chickens can also be observed in other birds infected with *A. fumigatus*, including pigeon (36), gosling (37), and quail (38).

*Aspergillus fumigatus* infection caused extensive infiltration of inflammatory cells containing heterophils and macrophages as early as 1 dpi in chickens, indicating that substantial conidia can lead to hyperacute infection of aspergillosis and elicit the innate immune responses. In the pulmonary diseases, pulmonary macrophages play a central role in protecting against *A. fumigatus* and are essential for regulating the pulmonary innate immune response to fungal infection (39). It has been reported that the load of *A. fumigatus* increased in alveolar macrophage-deficient mice (40). In addition, neutrophils are important cells to defense against various pathogens including *Aspergillus* in the lung (41). The ability of neutrophils to efficiently kill *A. fumigatus* hyphae *in vivo* has been demonstrated by the real-time visualization in the zebrafish (42). These results suggested that macrophages and heterophils are required for *A. fumigatus* infection in chickens.

To better understand the pathogenesis of *A. fumigatus* in chickens, it is necessary to explore the interaction between host and pathogen. Since the host innate immune response is essential for defending against *A. fumigatus* in the early infection, the mRNA expressions of TLR and pro-inflammatory cytokines were measured in this study. Multiple PRRs are involved in sensing fungi during infection. TLR including TLR1, TLR2, TLR4, and TLR9 are crucial for the recognition of fungal cell wall components and the resultant antifungal responses (28, 43, 44). Moreover, it is demonstrated that the TLR associated MyD88 signal pathway is required for controlling fungi in mice (45). In the present study, the expressions of TLR1 and TLR2 were significantly upregulated in the chicken infected lungs and spleen, especially the TLR2 mRNA, which was increased by 6.83-fold in the lungs at 3 dpi, which may indicate that both were involved in the recognition of *A. fumigatus* in chickens (46, 47). The activation of PRRs can trigger the production of a large number of downstream pro-inflammatory cytokines (48). Th1-type cytokines, such as IL-1, IL-12, TNF-α, IFN-γ, and some chemokines including MIP-1 and Cxcl-8 play an important role in antifungal infection (27). In this study, the expressions of most pro-inflammatory cytokines, such as IL-1β, Cxcl-8, IL-12, TNF-α, and IFN-γ were increased, meaning they likely facilitated the inflammatory response to *A. fumigatus* in chickens. Recently, Li et al. reported that *A. fumigatus* infection induced inflammatory responses characterized by the increased production of IL-1 and IL-12 in chicken macrophage cell line HD-11, and exposure of *A. fumigatus*-infected macrophages to T-2 toxin further upregulated the expression of IL-1L, IL-6, IL-12, and IL-18 (49). The upregulated expression of IL-1L and IL-12 in infected chickens was compatible with the *A. fumigatus*-induced production of these cytokines in mouse alveolar macrophage (50), but IL-18 change was unconsistent with that in mouse lung tissue (51). It is known that macrophages not only phagocytize pathogens, but they can also regulate the immune response and secrete cytokines, such as TNF-α and IFN-γ. It has been reported that *A. fumigatus* extract differentially regulated CD8+ T cells expansion accompanied by differentiation into IFN-γ-producing cytotoxic cells to promote host immunity, but had no effect on CD4+ T cells response (52). In the current study, the production of TNF-α and IFN-γ was significantly upregulated in infected chickens, it may act as a stimulator to induce classically macrophages and heterophils, destruct intracellular *A. fumigatus* and further promote a local Th1 environment (53). Cxcl-8 recruits neutrophils to the sites of inflammation and mediates the release of antimicrobial peptides (10). In *A. fumigatus*-infected chickens, Cxcl-8 expression reached the maximum value at 3 dpi, since the resistance to aspergillosis is dependent on heterophils (45), these data suggested that Cxcl-8 could recruit heterophils to fight against *A. fumigatus* infection in chickens, which is consistent with histopathological examination of a large number of heterophils in the lungs. However, heterophils functions as a double-edged sword, on the one hand, they are essential in acute inflammatory responses of *A. fumigatus* infection, but on the other hand, the excessive release of oxidants and proteases from heterophils may result in tissues injury.

On the whole, although the immune responses to *A. fumigatus* has been activated in chickens, they may not efficiently prevent the early establishment of infection in this study. As a result, many chickens died from *A. fumigatus* infection in this study. Part of the reason may be the inoculation method and dose, and part may be due to the fact that conidia are cytotoxic to macrophages (54), and the number of fungal conidia exceed the killing capacity of the host immunity, leading to intracellular germination, colonization, and disease, moreover excessive pathogen replication may further exacerbate deleterious inflammation. Additionally, considering the roles played by humoral factors in the host response to Aspergillus (55), further studies will explore the adaptive immune response to *A. fumigatus*.

## Conclusions

In summary, the typical clinical symptoms and histopathological lesions were reproduced after *A. fumigatus* infection by thoracic intra-air sacs injection in chickens, with active recruitment of macrophages and heterophils. *A. fumigatus* can trigger TLR mediated innate immune responses, leading to the massive production of IL-1e, TNF-α, Cxcl-8, and IFN-γ etc. pro-inflammatory cytokines in chickens.

## Data Availability Statement

All datasets generated for this study are included in the article/Supplementary Material.

## Ethics Statement

These animal experiments were approved by the Shandong Agricultural University Animal Care and Use Committee (SDAUA-2015-012) and performed according to the approved guidelines.

## Author Contributions

ZC and ML carried out the experiments and wrote the manuscript. YW performed the experiments and analyzed the data. TC designed the experiments. YC and NL designed the experiments, reviewed the manuscript, and approved the submission.

### Conflict of Interest

The authors declare that the research was conducted in the absence of any commercial or financial relationships that could be construed as a potential conflict of interest.

## Supplementary Material

Supplementary Figure 1 Gross lesions of air sacs and lungs infected with *Aspergillus fumigatus*. **(A)** At 1 dpi, slight thickness, turbidity and a small amount of yellow white exudate in air sac; **(B)** At 3 dpi, yellow white exudate increased in air sac; **(C)** At 5 dpi, a large amount of yellow white caseous exudate in air sac; **(D)** Clean and transparent in normal air sac; **(E)** At 1 dpi, focal edema and hemorrhage in the lung; **(F)** At 3 dpi, the lesions size increased, with edema and hemorrhage in the lung; **(G)** At 5 dpi, yellow white necrosis in the majority of lung; **(H)** Normal lung.
