# Supplementary figures and images for: Pathogenicity and Immune Responses of Aspergillus fumigatus Infection in Chickens
Source: Front Vet Sci. 2020 Mar 11;7:143. doi: 10.3389/fvets.2020.00143 (PMC7078108; doi:10.3389/fvets.2020.00143)

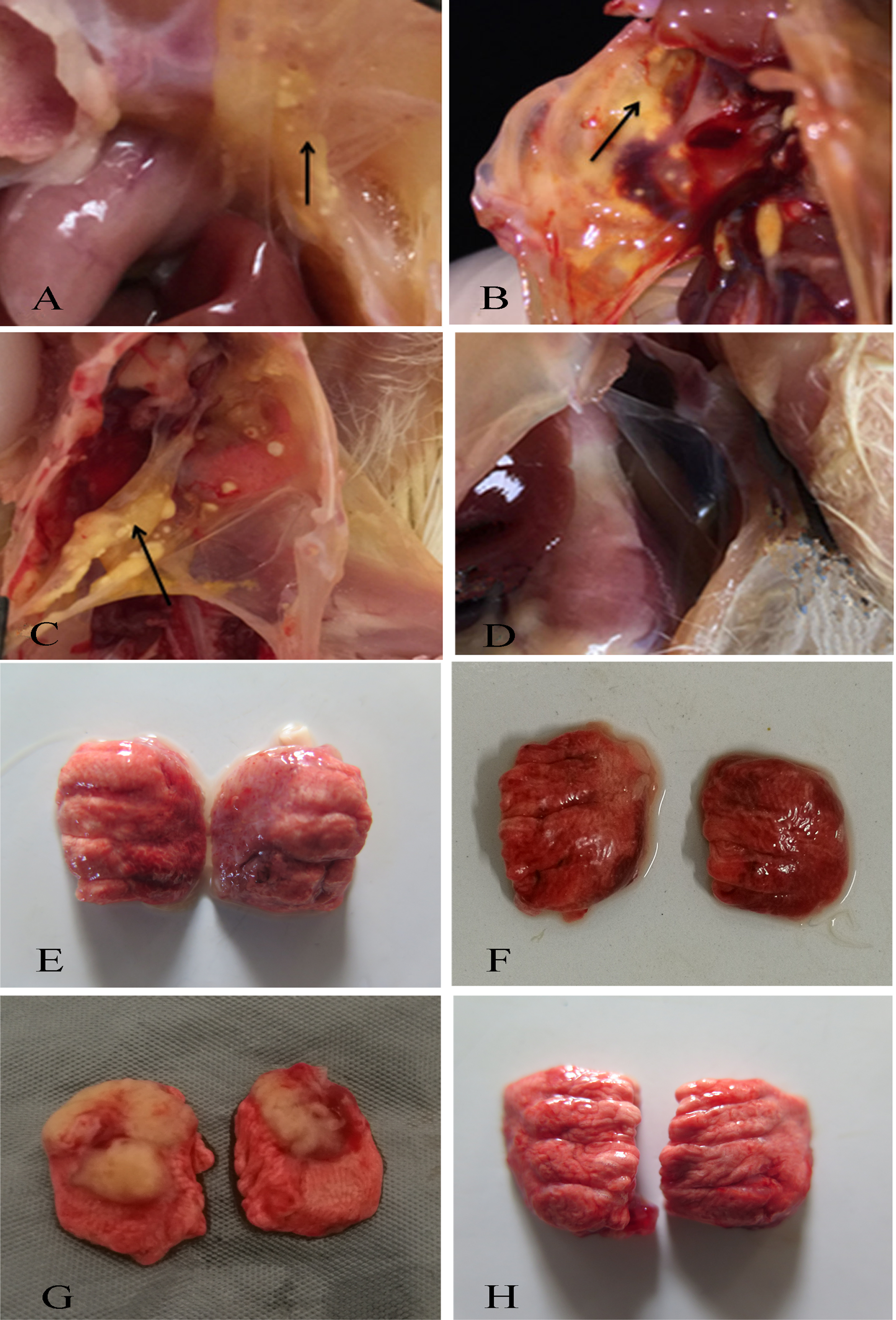

Supplement: Supplementary Figure 1 — Gross lesions of air sacs and lungs infected with Aspergillus fumigatus. (A) At 1 dpi, slight thickness, turbidity and a small amount of yellow white exudate in air sac; (B) At 3 dpi, yellow white exudate increased in air sac; (C) At 5 dpi, a large amount of yellow white caseous exudate in air sac; (D) Clean and transparent in normal air sac; (E) At 1 dpi, focal edema and hemorrhage in the lung; (F) At 3 dpi, the lesions size increased, with edema and hemorrhage in the lung; (G) At 5 dpi, yellow white necrosis in the majority of lung; (H) Normal lung. [file Image_1.TIF]
